# Contrasting biogeography and diversity patterns between diatoms and haptophytes in the central Pacific Ocean

**DOI:** 10.1038/s41598-018-29039-9

**Published:** 2018-07-19

**Authors:** Hisashi Endo, Hiroyuki Ogata, Koji Suzuki

**Affiliations:** 1 Faculty of Environmental Earth Science, Hokkaido University, North 10 West 5, Kita-ku, Sapporo, Hokkaido, 060-0810 Japan; 2 CREST, Japan Science and Technology, North 10 West 5, Kita-ku, Sapporo, Hokkaido, 060-0810 Japan; 3 Bioinformatics Center, Institute for Chemical Research, Kyoto University, Gokasho, Uji, Kyoto, 611-0011 Japan

## Abstract

Diatoms and haptophytes are two major phytoplankton groups, playing pivotal roles in global biogeochemical cycles and marine ecosystems. In general, diatoms have higher growth rates than haptophytes, whereas haptophytes tend to have higher nutrient uptake affinity. However, precise linkages between their ecological traits and geographical distributions remain poorly understood. Herein, we examined the basin-scale variability of the abundance and taxonomic composition of these two phytoplankton groups across 35 sites in the Pacific Ocean using DNA metabarcoding. The diatom community was generally dominated by a few genera at each sample site, whereas the haptophyte community consisted of a large number of genera in most of the sites. The coexistence of various haptophyte genera might be achieved by diversification of their ecophysiological traits such as mixotrophy. On the other hand, the diatom community might experience greater inter-genus competition due to the rapid uptake of nutrients. Our data further supports the notion that their distinct ecological strategies underlie the emergence of contrasting diversity patterns of these phytoplankton groups in the central Pacific at a basin scale.

## Introduction

The largest habitat on Earth is the pelagic region of the Pacific Ocean, which covers approximately half of the global ocean. Biological characterization of such large oceanic provinces is essential to assess the productivity and stability of marine ecosystems and to predict the anthropogenic effects on these systems^1,2^. The biogeography of phytoplankton is of primary interest because these organisms regulate the primary productivity of oceans, and therefore, the global nutrient cycles and fishery production^3,4^.

Numerous studies have examined the spatial contrasts in community composition and diversity of phytoplankton using field observations, satellite remote sensing, and ecological modeling^5–8^. However, most of these studies have assessed communities at the phylum or functional group level despite the large diversity of phytoplankton species. Recent advances in high-throughput DNA sequencing (i.e., the next-generation sequencing; NGS) technologies now allow us to analyze a large number of samples in parallel at the DNA sequence level, and consequently, large-scale community data sets can be relatively easily generated at a finer scale than before. Using these technologies, global surveys of marine plankton (e.g., the *Tara* Oceans expedition) have revealed much higher diversity of phytoplankton than previously recognized^9,10^.

Among the oceanic phytoplankton groups, diatoms and haptophytes have been particularly well investigated. Diatoms are distributed ubiquitously in the aquatic environment and are thought to be responsible for approximately 40% of the total oceanic primary production^11^ and particulate carbon exported to depth as part of the biological pump^12^. Haptophytes are also ubiquitous in the euphotic layers of the world oceans and are important contributors to marine primary production^13^. de Vargas *et al*.^9^ revealed that diatoms and haptophytes have particularly high richness in operational taxonomic units among phytoplankton groups using ribosomal DNA metabarcoding. It is also noted that diatoms and haptophytes are experiencing competitive interactions in natural environments, and the results of competition between the two algal groups can potentially influence the biogeochemical processes in the ocean^14,15^.

Despite the comparable importance of diatoms and haptophytes in biogeochemical and ecological processes, these phytoplankton probably adopt different strategies for growth. Ecologically, diatoms are generally *r*-strategists, characterized by rapid growth rates under suitable conditions^16,17^. By contrast, haptophytes are likely *K*-strategists^17,18^, which are characterized by slow growth rates, although some species, such as *Emiliania huxleyi* and *Phaeocystis antarctica*, can form blooms^19,20^. In general, *r*-selected taxa allocate larger proportion of resources to reproduction, whereas *K*-selected taxa allocate less resources to reproduction and more to other activities that ensure the survival of individuals, resulting in maintaining a constant population size. Consequently, the former have competitive advantage in changing (less stable) environments, whereas the latter favor habitats with a long durational stability^21^.

A clear separation of *r*- and *K*-tradeoffs between diatoms and haptophytes was also observed in a metatranscriptomic study in an oligotrophic ocean^18^, where diatoms were found to increase growth-related transcriptional activity with nutrient enrichments, whereas the activity of haptophytes was decreased. The difference in ecological strategies between diatoms and haptophytes may also affect their global distribution and diversity patterns. However, to our knowledge, no study has examined how the distinct ecological strategies contribute to the shaping of their biogeography on a large scale.

**Figure 1:**
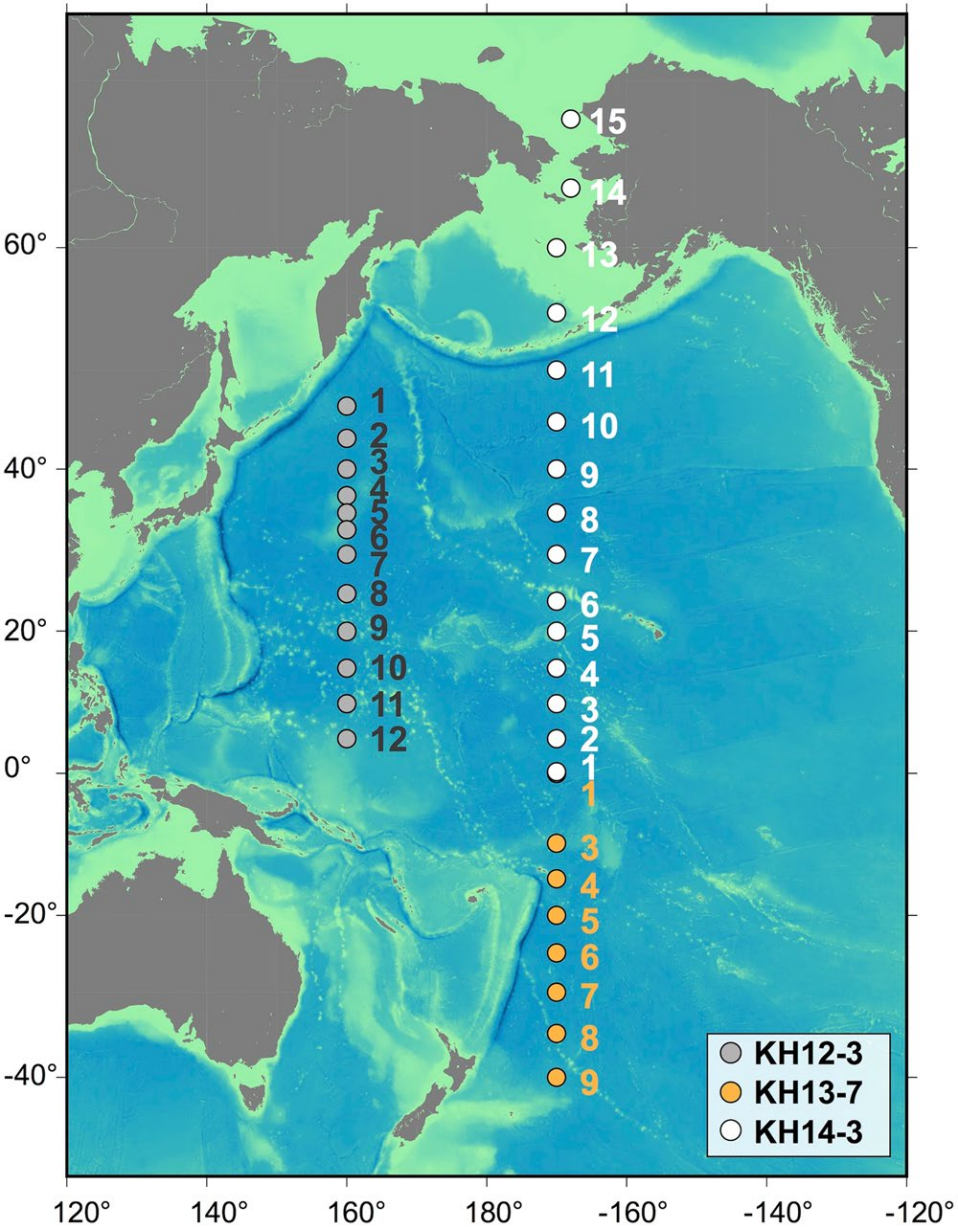
Map of the sampling sites. Gray, yellow, and white circles denote the sampling locations during the KH-12-3, KH-13-7, and KH-14-3 cruises. The base figure was created using Generic Mapping Tools (GMT) version 5.4.1 (https://www.soest.hawaii.edu/gmt/).

In this study, we conducted field sampling at 35 sites, covering a wide area of the central Pacific Ocean and the Bering and Chukchi Seas (Fig. 1). Three cruises were conducted along 160°E and 170°W transects during 2012–2014 (Supplementary Table 1). Using quantitative polymerase chain reaction (qPCR) and NGS techniques, we assessed the abundance, diversity, and community composition of diatoms and haptophytes to establish their ecological realms in the Pacific Ocean and to investigate the underlying ecological forces shaping the biogeography of these phytoplankton.

## Results

### Abundance of diatoms and haptophytes and their environmental relationships

**Figure 2:**
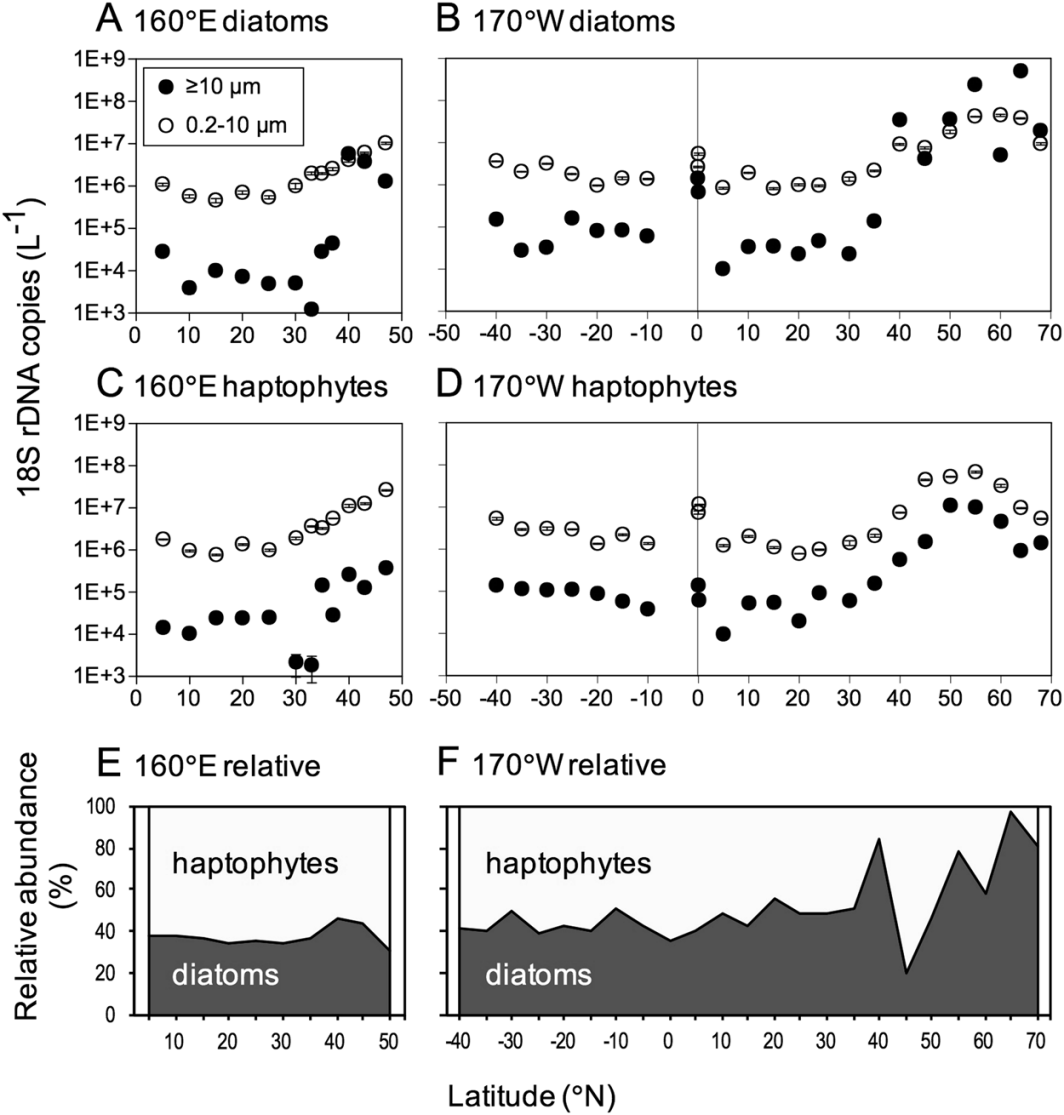
Meridional (South–North) variation in diatom and haptophyte abundances in the surface layer. Upper (**A** and **B**) and middle (**C** and **D**) graphs show the abundance of size-fractionated 18S rDNA in diatoms and haptophytes, respectively. Left (**A** and **C**) and right (**B** and **D**) graphs indicate the abundance along the 160°E and 170°W transects, respectively. Error bars denote ±1 standard deviation (SD, n = 3). Lower graphs (**E** and **F**) show the relative abundance of 18S rDNA from diatoms and haptophytes along the 160°E and 170°W transects, respectively.

The abundance of diatoms and haptophytes, assessed by copy numbers of 18S rRNA gene (hereafter, referred to as 18S rDNA), showed clear meridional gradients in both surface and deep chlorophyll maximum (DCM) layers. For both diatoms and haptophytes, the abundances increased significantly at high latitudes, although peaks of abundance differed between diatoms and haptophytes (Fig. 2 for surface, Supplementary Fig. S1 for DCM). At high latitudes (≥55°N and 40°N for surface and ≥60°N and 40°N for DCM), diatoms dominated over haptophytes, whereas the abundance of diatoms and haptophytes was comparable at the other locations.

The abundance of diatoms reached a maximum at 47°N in both the surface (Fig. 2A) and DCM layers (Supplementary Fig. S1A) on the 160°E transect, whereas it was remarkably increased north of 40°N on the 170°W transect and reached a maximum at 64°N in both the surface and DCM layers (Fig. 2B, Supplementary Fig. S1B). Between the two size fractions, the abundance of diatoms was higher for small-sized fractions (0.2–10 µm) than for large-sized fractions (≥10 µm), except at a few sites north of 40°N. The abundance of diatoms was negatively correlated with temperature and salinity in both the surface and DCM layers (Pearson’s test, *p* < 0.01; Table 1). However, the abundance of diatoms was not correlated with the concentrations of nitrate, ammonia, phosphate and silicate.

**Table 1:** Pearson correlations between environmental/biological variables and abundance or Shannon index (*H*′) of 18S rDNA in diatoms and haptophytes (n = 35 or 31).

		Temp	Sal	NO_3_	NH_4_	PO_4_	Si(OH)_4_	18S rDNA copy
Diatoms								
Abundance	Surface	−0.48**	−0.59**	**−**0.02	**−**0.03	0.21	**−**0.02	—
	DCM	−0.40*	−0.52**	0.03	0.21	0.26	0.06	—
Diversity (*H*′)	Surface	**−**0.24	**−**0.09	**−**0.04	0.03	0.06	**−**0.07	0.19
	DCM	0.16	0.08	**−**0.34	**−**0.05	**−**0.30	**−**0.24	0.11
Haptophytes								
Abundance	Surface	−0.65**	−0.64**	0.60**	0.04	0.77**	0.69**	—
	DCM	−0.47**	−0.54**	0.75**	0.05	0.74**	0.90**	—
Diversity (*H*′)	Surface	0.80**	0.72**	−0.53**	−0.55**	−0.69**	−0.51**	−0.60**
	DCM	0.72**	0.73**	−0.53**	−0.48**	−0.69**	−0.50**	−0.47**

Abbreviations: Temp, temperature; Sal, salinity; NO_3_, nitrate; NH_4_, ammonia; PO_4_, phosphate; Si(OH)_4_, silicate. *P*-value < 0.05 and <0.01 are marked with * and ** in bold text, respectively.

The abundance of haptophytes increased with latitude, and the values reached maxima at 47°N in both the surface and DCM waters on the 160°E transect (Fig. 2C, Supplementary Fig. S1C). The abundance of haptophytes also increased exponentially with latitude along the 170°W transect, and reached the maximum values at 55°N in both the surface and DCM layers (Fig. 2D, Supplementary Fig. S1D). The abundance of haptophytes was generally much higher for small-sized fractions than for large-sized fractions, except in the DCM layer at 68°N. The abundance of haptophytes was negatively correlated with temperature and salinity, and positively correlated with the concentrations of nitrate, phosphate, and silicate (Pearson’s test, *p* < 0.01; Table 1).

### Distinct diversity patterns between diatoms and haptophytes

**Figure 3:**
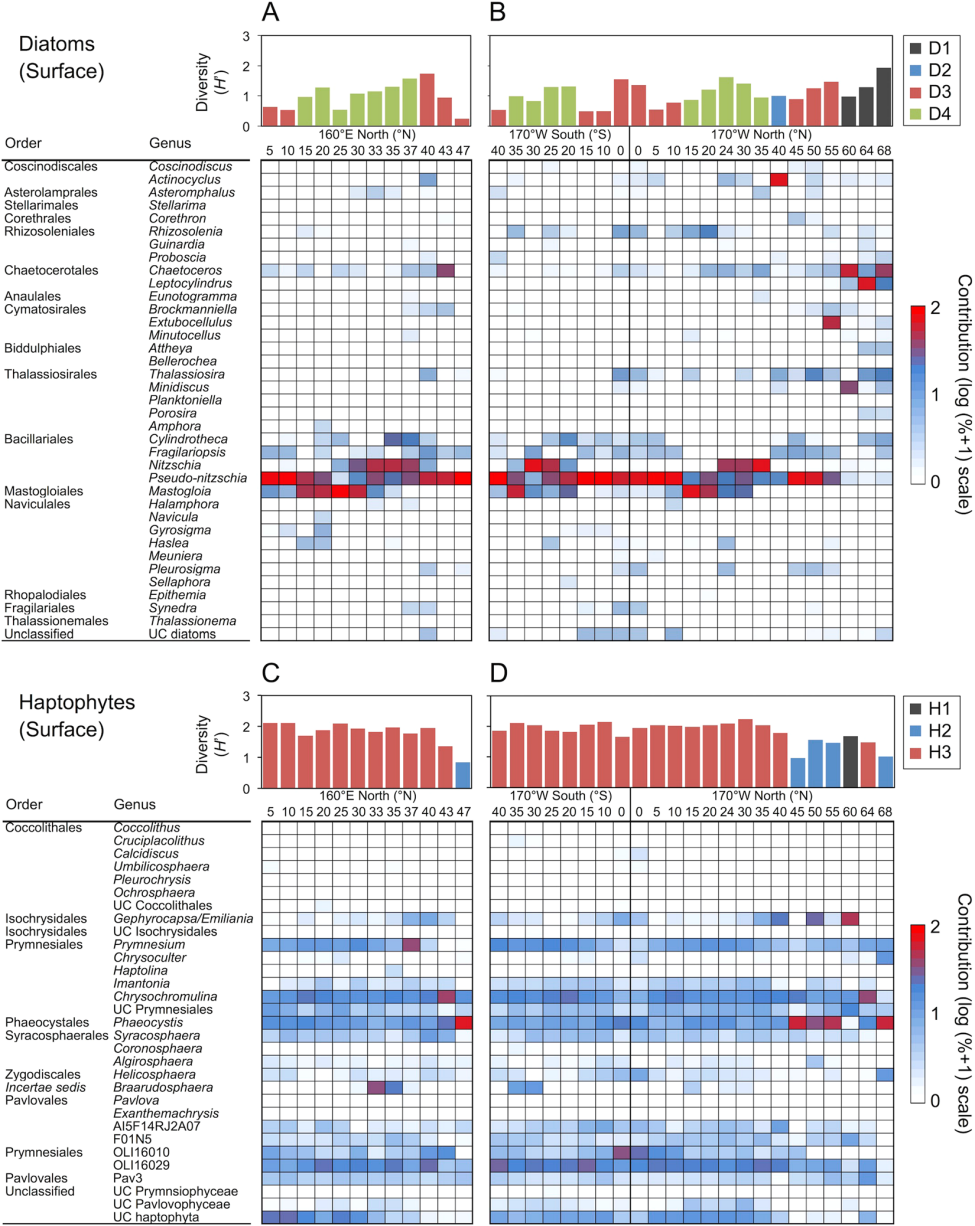
Diversity and distribution patterns of diatoms (**A** and **B**) and haptophytes (**C** and **D**) in the surface layer. Left (**A** and **C**) and right (**C** and **D**) graphs represent the data collected along the 160°E and 170°W transects, respectively. Graphs for diatoms and haptophytes show data from the total (>0.2 µm) and small (0.2–10 µm) fractions, respectively. The scale bars are color-coded based on the biogeographical classification defined in Fig. S3. Diatom genera accounting for at least 1% of the total community in at least one of 66 sampling events are shown in the heatmaps.

The diatom-specific 18S rDNA sequences were classified into 51 phylogenetic groups, which were composed of 50 diatom genera and unclassified diatoms. The haptophyte 18S rDNA sequences were classified into 31 phylogenetic groups (25 were taxonomically assigned at the genus or putative clade level, three at the order level, two at the class level, and the remaining group was annotated as an unclassified haptophyte). The diatom community was generally dominated by a few genera across the sites, whereas the haptophyte community contained a variety of genera in most of the sites (Fig. 3, Supplementary Fig. S2).

**Figure 4:**
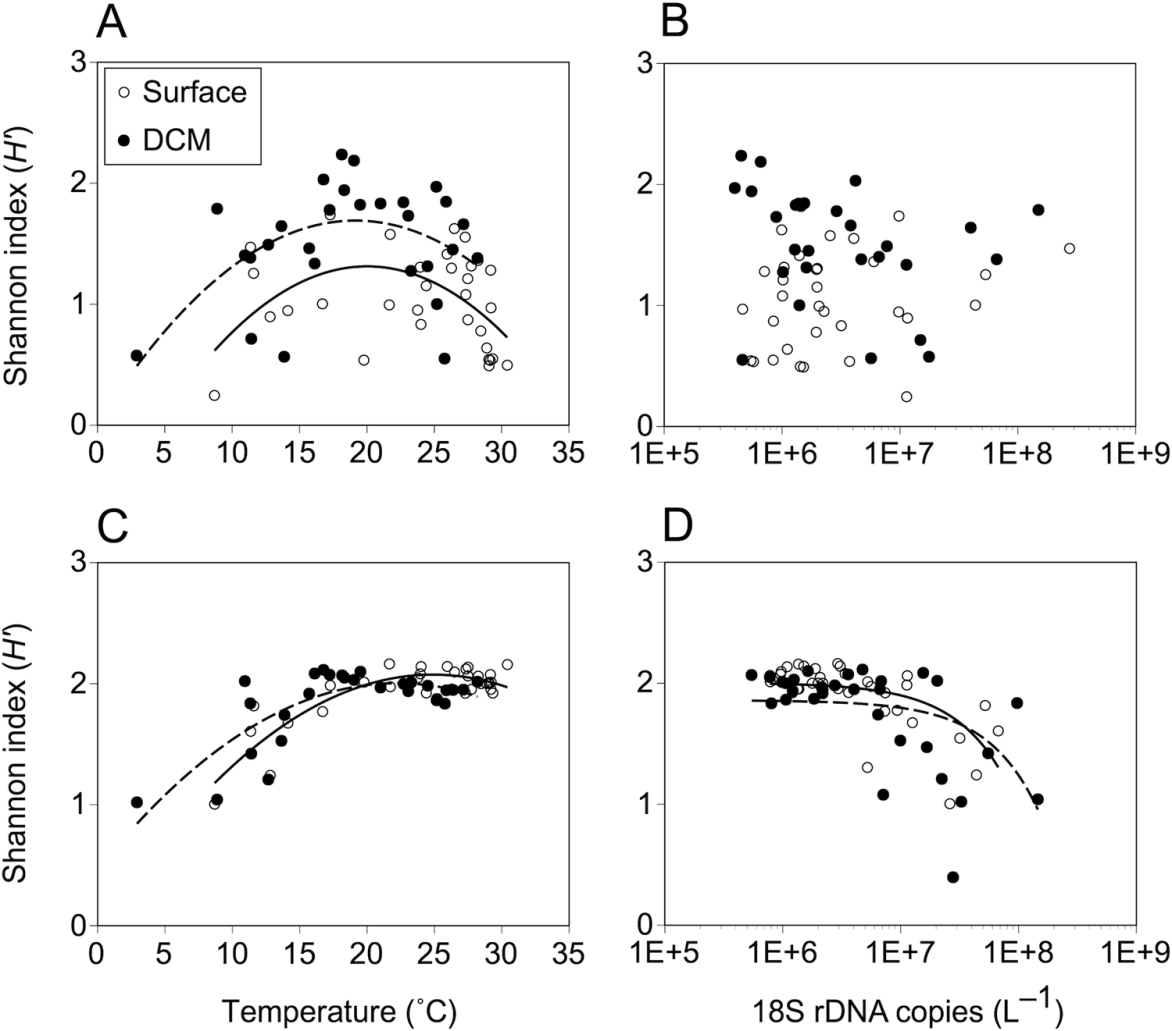
Relationships between phytoplankton diversity and temperature (**A** and **C**) and 18S rDNA copy abundance (**B** and **D**). Upper (**A** and **B**) and lower (**C** and **D**) graphs indicate data from diatoms and haptophytes, respectively. Solid and dashed lines represent regression fits of surface and DCM layers, respectively (n = 32 or 28). The sample sites in the polar biomes (60–68°N) were excluded from the analysis.

The diversity of diatoms, measured by Shannon’s *H*′, varied considerably among the geographic locations. For the surface waters, latitudinal gradients in diatom diversity were observed along both the 160°E and 170°W transects (Fig. 3). In surface waters, the genus-level diatom diversity tended to increase around 35°–40°N, and the highest *H*′ value was observed at 40°N (*H*′ = 1.60; Fig. 3A) along the 160°E transect. On the 170°W transect, the diatom diversity peaked near the equator, subtropical (20–25°S and 24–30°N), subpolar (55°N), and polar (68°N) regions in the surface layers, whereas the diversity of diatoms was distributed more randomly in the DCM layer (Fig. 3B, Supplementary Fig. S2B). The diversity of diatoms was not significantly correlated with any environmental or biological variable in both the surface and DCM layers (Pearson’s test, *p* > 0.05; Table 1). However, in the case when the neritic Bering and Chukchi Seas were excluded from the dataset, significant parabolic relationships were found between the diatom diversity and temperature, and the diversity peaked at approximately 20 °C in both the surface and DCM layers (MOS test, *p* < 0.05; Fig. 4A). Neither a linear nor a polynomial relationship was detected between diatom diversity and abundance (*p* > 0.05; Fig. 4B).

The diversity of haptophytes was higher than diatom diversity and remained relatively constant (*H*′ = ca. 2.0) at most of the sites to the south of 35°N in both the surface and DCM layers along the two transects, although the diversity tended to decrease at higher latitudes in the northern hemisphere (Fig. 3, Supplementary Fig. S2). The levels of haptophyte diversity were positively correlated with temperature and salinity in both the surface and DCM layers and negatively correlated with the concentrations of nitrate, ammonia, phosphate, and silicate (Pearson’s test, *p* < 0.01; Table 1). A significant parabolic correlation was also found between haptophyte diversity and temperature, and the diversity reached a maximum at 20–30 °C in both the surface and DCM layers (MOS tests, *p* < 0.05; Fig. 4C). The haptophyte diversity was negatively correlated with their abundance in both the surface and DCM layers (Pearson’s test, *p* < 0.01; Fig. 4D).

### Meridional gradient of community composition differs between diatoms and haptophytes

**Figure 5:**
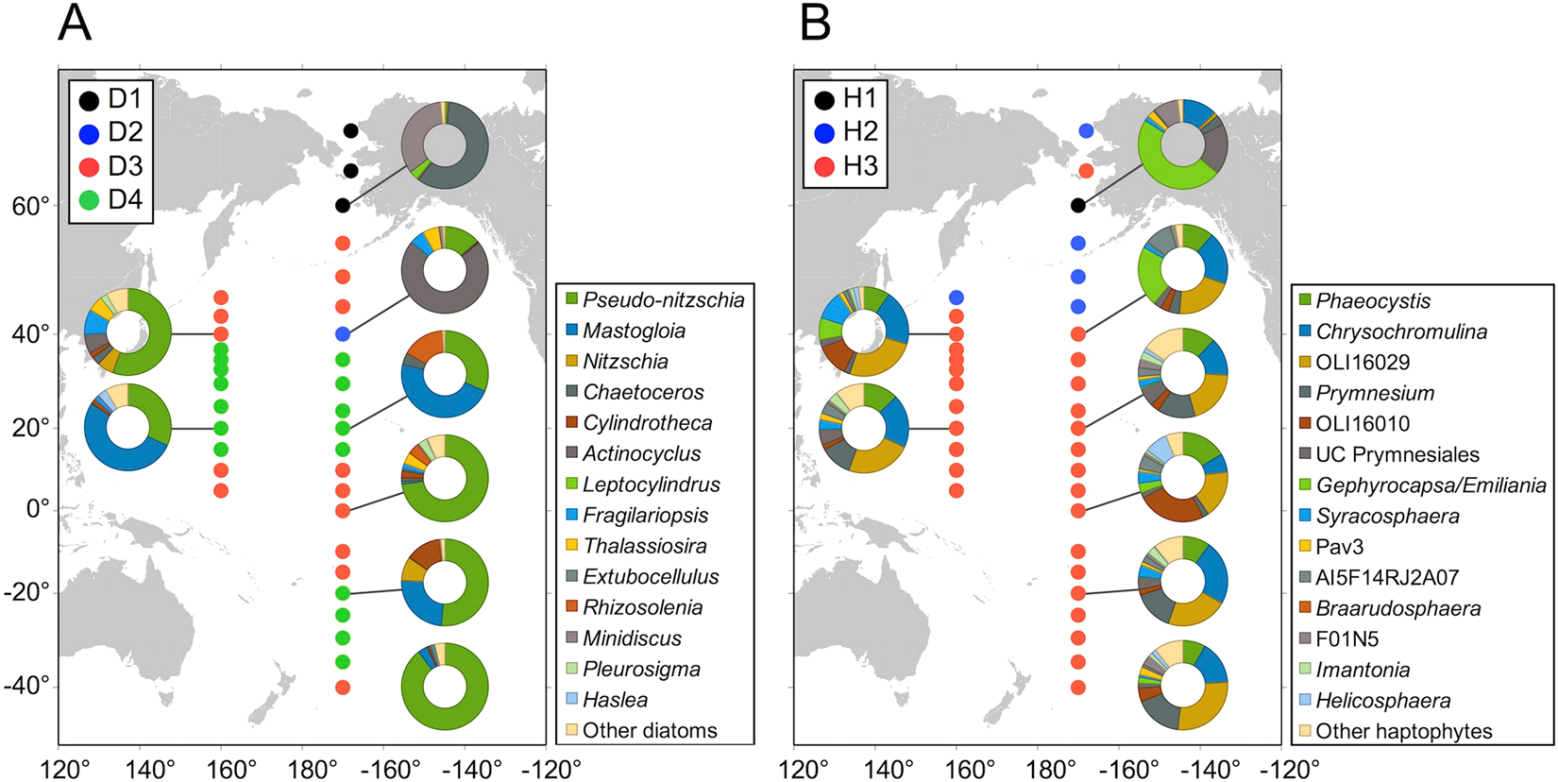
Biogeographical classifications of the study area based on the similarity of community compositions of diatoms (**A**) and haptophytes (**B**). Dots of the identical color indicate locations with the identical group. The sampling months are shown in the figures. Pie charts indicate the relative contribution of each genus to the total at 40°N and 20°N on the 160°W transect, and 60°N, 40°N, 20°N, 0°N, 20°S and 40°S on the 170°W transect. The base figure was created using GMT version 5.4.1.

Based on the community composition, the diatom communities were classified into four major groups for the surface layer (Groups D1–D4; Supplementary Fig. S3A) and for the DCM layer (Groups D5–D8; Supplementary Fig. S3B). The distribution of these groups showed a clear spatial pattern, defining ecological realms of the diatom communities (Fig. 5A, Supplementary Fig. S4A). Differences in the diatom community composition among groups were verified by permutational multivariate analysis of variance (PERMANOVA, *p* < 0.001). The diatom Group D1 (located in the arctic region) was primarily composed of centric diatom genera, *Chaetoceros* (an average contribution of 35.4% to the total diatom community), followed by *Leptocylindrus* (29.8%), and *Minidiscus* (12.6%) (Supplementary Table S2). Groups D2 (subtropical-subarctic transition) and D3 (tropical and subarctic) were characterized primarily by the dominance of a centric genus *Actinocyclus* and a pennate genus *Pseudo-nitzschia*, respectively (contributions of 72.0% and 75.4%, respectively). Group D4 (subtropical) was further divided into two clusters D4-1 and D4-2 (PERMANOVA, *p* < 0.001), which were marked by the dominance of the raphid pennate diatom genera *Nitzschia* (53.3%) and *Mastogloia* (55.7%), respectively. For diatoms in the DCM layer, Group D5 (arctic) was composed of a mixture of centric diatom genera, *Thalassiosira* (32.8%), *Leptocylindrus* (25.9%), *Chaetoceros* (16.5%), and *Porosira* (11.3%). The genus *Minidiscus* was dominant (58.6%) in Group D6 (arctic), which was recorded at only one sample location. The genera, *Pseudo-nitzschia* (31.4%), *Amphora* (pennate, 30.8%), and *Eunotogramma* (centric, 10.2%), were important contributors to Group D7 (subtropical and subtropical-subarctic transition in the southern hemisphere), whereas the genus *Pseudo-nitzschia* was predominant (59.1%) in Group D8 (subtropical to subarctic).

The haptophyte communities were clustered into three major groups in the surface layers (Groups H1–H3; Fig. 5B, Supplementary Fig. S3C) and four groups in the DCM layer (Groups H4–H7; Supplementary Figs S3D and S4B). The grouping was also verified with PERMANOVA (*p* < 0.001). The distribution pattern of the haptophyte groups in the surface layer depended on the sample locations to some extent: the subarctic and arctic regions (Groups H1 and H2) and between tropical and subarctic regions (Group H3). Group H1 at the surface was recorded at only one sample location (40°N), and was dominated by the genus *Gephyrocapsa*/*Emiliania* (47.9%), followed by unclassified Prymnesiales (18.5%) and *Chrysochromulina* (12.2%) (Supplementary Table S2). Group H2 was mostly dominated by the genus *Phaeocystis* (54.9%). Group H3, which included 29 sample stations, was composed of a mixture of OLI16029 (18.0%), *Chrysochromulina* (17.3%), *Phaeocystis* (11.8%), and *Prymnesium* (10.3%).

Groups H4 and H5 in the DCM layers were characterized by the dominance of *Phaeocystis* (69.3%) and *Chrysochromulina* (64.8%), respectively. Group H6 was dominated primarily by uncultured haptophyte, OLI16010 (21.6%), followed by OLI16029 (16.3%) and *Phaeocystis* (14.7%). Group D7 was primarily composed of *Gephyrocapsa*/*Emiliania* (36.4%), followed by *Phaeocystis* (17.4%) and *Chrysochromulina* (13.3%).

### Distribution of *Braarudosphaera* correlates with that of diazotrophic symbiont, UCYN-A1

For haptophyte community Group H3, the genus *Braarudosphaera* was an important contributor (10.5–35.2%) to the haptophyte assemblages between 33°N and 35°N along the 160°E transect and between 30°S and 35°S along the 170°W transect, but the genus was rarely detected in the other samples of Group H3 or in other community groups. The endemic occurrence of *Braarudosphaera* was further supported by the distribution of nitrogenase gene (*nifH*) of the nitrogen-fixing cyanobacterium, UCYN-A1, likely associated with *Braarudosphaera bigelowii*^22,23^, and 18S rDNA of the UCYN-A1 host (Supplementary Fig. S5). Consistent with the distribution of *Braarudosphaera*, the UCYN-A1 *nifH* gene and its host 18S rDNA were observed exclusively in the surface layers, whereas they were rarely detected in the DCM layers.

The copy number of the UCYN-A1 *nifH* gene was positively correlated with that of the UCYN-A1 host 18S rDNA and that of *Braarudosphaera*-derived 18S rDNA, which was calculated by multiplying the haptophyte 18S rDNA abundance by the relative abundance of *Braarudosphaera* measured by metabarcoding (Pearson’s test, *p* < 0.01; Supplementary Fig. S5).

### Environmental variables explaining the community composition

**Figure 6:**
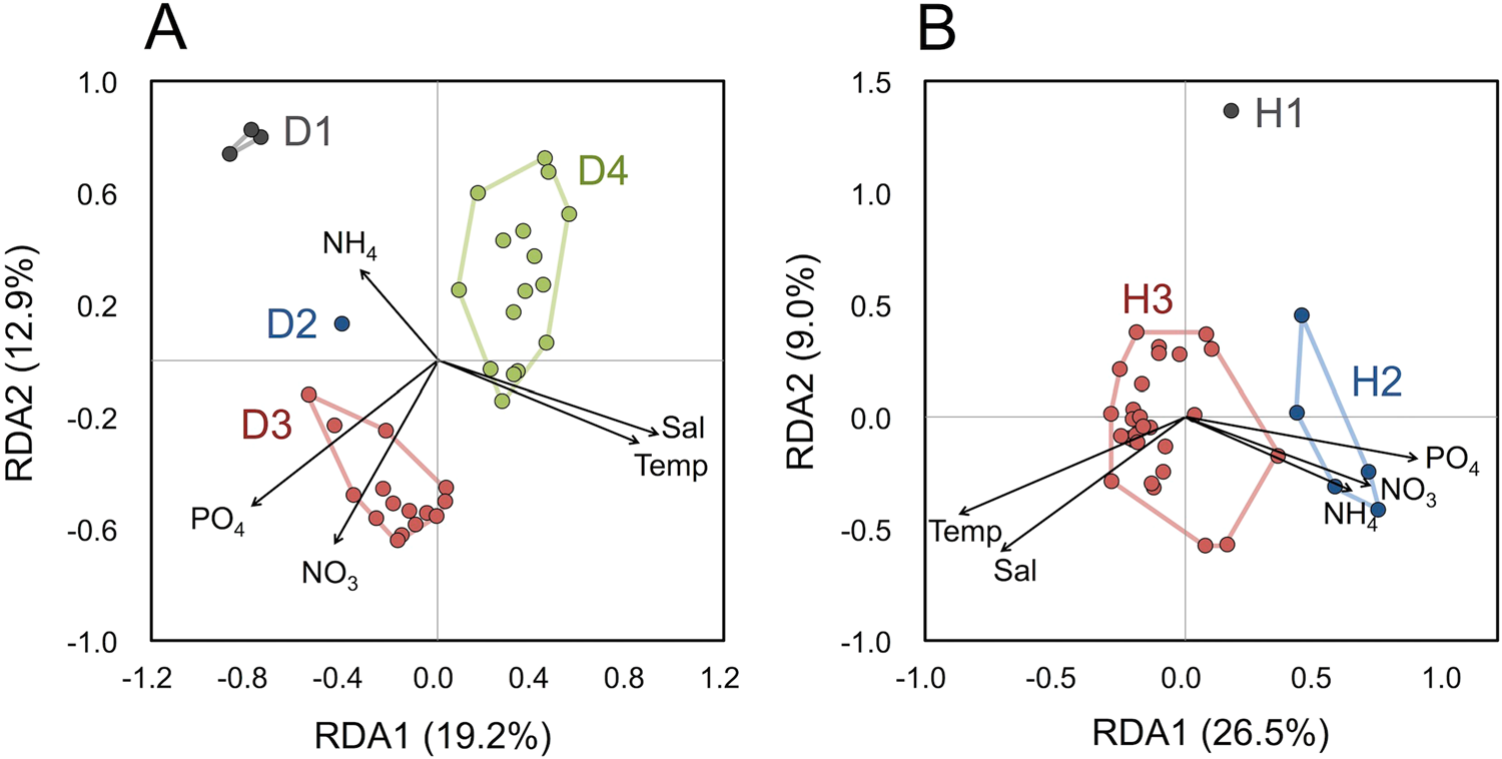
Redundancy analysis (RDA) ordinations for environmental variables and community compositions of diatoms (**A**) and haptophytes (**B**) in the surface layer. The black arrows indicate the vectors of the explanatory variables. The sample events contained in each cluster (Supplementary Fig. S3) are distinguished by different colors.

Based on forward selection in the redundancy analysis (RDA), the variation of diatom community composition was explained primarily by salinity and phosphate in the surface layers and by salinity, nitrate, and phosphate in the DCM layers (*p* < 0.01; Fig. 6, Supplementary Fig. S6). The total variation constrained by the environmental variables was 38.8% for diatoms in the surface and 34.3% for those in the DCM layers.

The variation in haptophyte community composition at the surface was explained significantly by phosphate, followed by temperature and ammonia, whereas the variation in the DCM layer was explained primarily by phosphate, followed by salinity and nitrate (Forward selection, *p* < 0.01). The combination of the environmental variables explained 45.9% and 56.2% of the total variance in the surface and DCM layers, respectively.

## Discussion

A comparison of the abundance of each taxon revealed that diatoms dominated over haptophytes at stations north of the Aleutian Islands (55°–68°N), whereas haptophytes outcompeted diatoms south of the Aleutian Islands (45°–50°N) (Fig. 2F, Supplementary Fig. S1F). These distribution patterns were consistent with those estimated on the basis of phytoplankton pigment signatures^7,24^. It is known that the abundance of 18S rDNA fragment correlates significantly with the biovolume across a wide variety of eukaryotes^9^. Thus, our results can be indicative of resource competition between diatoms and haptophytes. According to the theory of *r-K* selection^13^, diatoms evolved as *r*-strategists, which specialize with maximum growth rates under nutrient-replete and highly disturbed environments^17^. This trait would enable diatoms to dominate in high-latitude ecosystems. To the south of 40°N, the abundance of diatoms and haptophytes was relatively low, and the contribution of haptophytes tended to increase slightly. These regions are characterized by low macronutrient availability (Supplementary Table 1), which is caused by strong stratification in the water column. Because haptophytes, especially in small cells, are generally expected to have higher affinity for nutrients (*K*-strategist)^18,25^, they might be more adapted to low nutrient and turbulent conditions.

The latitudinal trend in the abundance of diatom and haptophyte was fairly consistent between the surface and DCM layers. In most of the stations, the DCM layers were found around the depth of euphotic layer, and those were usually deeper than the mixed layer depth (MLD) (Supplementary Table 1). Additionally, a strong correlation was detected between the depths of the DCM and euphotic layers (Fig S7), whereas there was no significant correlation between the DCM depth and MLD. These results suggest that light availability was a key determinant for the DCM layer depths at a wider scale. However, the depths of the DCM layer were much shallower than those of the euphotic layer around the subarctic stations along the 170°W transect (40°–50°N), and the chlorophyll *a* biomass in these regions were relatively low despite the high levels of nutrient availability. This suggests that the water masses at these stations would be characterized as high-nitrate, low-chlorophyll (HNLC) waters, likely because of the low availability of iron^26^. Therefore, iron might be an important element for the competition of diatoms and haptophytes at the south of the Aleutian Islands, although iron data are unavailable in this study. Assuming that diatoms have higher Fe cellular quota and Fe half saturation constants than haptophytes^27^, haptophytes may have a competitive advantage in low iron waters over diatoms (especially, the centric species). The different iron uptake can also explain the positive correlation observed with nitrate for haptophyte abundance but not for diatoms (Table 1). Additionally, the intracellular nitrate pools in diatoms^28^ might mask the correlation between the abundance of diatoms and nitrate concentration in seawater. In other words, since there is a two-way feedback relationship between phytoplankton biomass and nutrient concentration, the lack of correlation with macronutrients could be due to the rapid nutrient uptake in diatoms^29^.

Differential loss rates due to top-down zooplankton grazing or viral attack might also be an important factor affecting the relative abundance of diatoms and haptophytes. For example, Nejstgaard *et al*.^30^ demonstrated that the growth of diatoms was significantly reduced by microzooplankton grazing, whereas that of *E. huxleyi* was unaffected in a mesocosm experiment. Therefore, zooplankton grazing can significantly control the abundance and community composition of phytoplankton via selective feeding. There is also growing evidence suggesting that viral infection can control phytoplankton population size, although only limited studies have been reported to date^31,32^.

Diatom assemblages in the Bering and Chukchi Seas (Groups D1, D5, and D6) were substantially different from those in the other study sites and consisted primarily of centric diatoms, such as *Chaetoceros* and *Thalassiosira* (Supplementary Table S2). These regions are located in the polar biome^33^ and were characterized by shallow depths (37–67 m) and high diatom abundance in this study (Fig. 2, Supplementary Table S1). An important source of nutrients for these regions is likely the nutrient-rich waters upwelled onto the Bering Sea shelf, which are transported through the Bering Strait to the Chukchi Sea shelf^34^. However, it has been reported that most nutrients in this region are exhausted due to the phytoplankton bloom composed mainly of diatoms and haptophytes during late spring and early summer^35^. Consequently, remineralized nutrients, such as ammonia, released from sediments of the Bering and Chukchi Sea shelves could contribute to the phytoplankton productivity in summer^34,36^. The ordinations of RDA in our study showed that the high latitude community groups, D1 and D5, were positively correlated with ammonia concentrations in both the surface and DCM layers, suggesting that regenerated nutrients contributed to the phytoplankton productivity. Because ammonia is also released from grazers^37^, top-down control might also contribute to the phytoplankton structure including diatoms.

In the open ocean biomes, raphid pennate diatoms, such as *Pseudo-nitzschia*, *Nitzschia*, and *Mastogloia*, predominated (Groups D3, D4, and D8). Although almost linages in raphid diatoms are benthic, some species in these genera have adapted to the planktonic life^38,39^. In evolution, the raphid pennate genera emerged at the Eocene/Oligocene boundary, when increased oxygen concentration decreased the iron availability in the open ocean^40^. Despite the short evolutionary history, raphid pennates have become the most diverse lineage among diatoms in the world today^38^. An important characteristic of raphid pennates is the raphe slits, which enable motility^41^. Kooistra *et al*.^38^ suggested that the acquisition of raphe opened the ecological niches previously unavailable to diatoms, which resulted in rapid diversification of this lineage in benthic habitats. However, our sequence data demonstrated that some raphid pennates were well adapted to the current open ocean environment. In particular, the genus *Pseudo-nitzschia* was ubiquitous and frequently dominated the total diatom communities in the central Pacific during summer. Previous studies demonstrated that the oceanic *Pseudo-nitzschia* species possessed higher iron-use efficiency than that of coastal diatoms^42^, and members of the genus can produce the iron storage protein, ferritin^43^. Therefore, enhanced iron-assimilation efficiency might enable these raphid pennates to outcompete centric diatoms in the open ocean where macronutrients are limited.

In contrast to diatoms, the community composition of haptophytes was relatively uniform over wide areas in the central Pacific Ocean (Fig. 3, Supplementary Fig. S2). Consequently, the community-based biogeographical classifications of haptophytes differed significantly from those of diatoms in both the surface and DCM layers (Fig. 5, Supplementary Fig. S4). The prevailing haptophyte Groups H3 and H6, which were distributed between the tropical and subarctic regions, were mostly composed of non-calcifying haptophyte lineages that were placed in the order Prymnesiales or Phaeocystales. A previous study also found that non-calcifying species were predominant among haptophytes in terms of diversity and abundance in the open ocean^24^. An important feature of the Prymnesiales is that the lineage possesses well-evolved haptonema, with which they catch organic particles and organisms as nutrient and energy sources^44^. Indeed, Groups H3 and H6 were associated with environments with relatively low nutrient concentrations (Fig. 6B, Supplementary Fig. S6B). Therefore, heterotrophic nutrient uptake processes might be important in nutrient-depleted environments.

For haptophytes at high latitudes, specific genera, such as *Phaeocystis*, *Chrysochromulina*, and *Gephyrocapsa*/*Emiliania*, dominated in terms of 18S rDNA abundance in both the surface and DCM layers (Fig. 3, Supplementary Fig. S2), and these sample locations were clustered into small groups (Supplementary Fig. S3). The RDA forward selection suggested that phosphate concentration was the primary factor affecting haptophyte community composition in both the surface and DCM layers, and was positively associated with Groups H2 and H4, which were dominated by *Phaeocystis* in the surface and DCM layers, respectively (Supplementary Table S2). Our findings may be indicative of high variability for phosphate uptake affinity among haptophyte species.

Notably, the calcareous haptophyte, *Braarudosphaera*, was restricted to only a few locations at the surface in the transition region (Fig. 3). The nitrogen-fixing cyanobacterium, UCYN-A1, is known to be obligatorily associated with *B. bigelowii*^23,45^ or its relatives^46^. Interestingly, the *nifH* gene derived from the UCYN-A1 was also abundant in these sites^47^. Additionally, we showed that the abundances of *Braarudosphaera* metabarcodes and the UCYN-A1 host rDNA, examined by qPCR, were strongly correlated with that of *nifH* gene derived from UCYN-A1 (Fig. S5). The symbiotic relationship between *B. bigelowii* and UCYN-A has been detected off southern California using an NGS-based approach^48^. Our result, thus, demonstrates the symbiotic association between UCYN-A1 and its hosts also occurring in the oceanic region of the Pacific. However, there is no observation for the existence of the calcareous cells of *B. bigelowii* in the open ocean, to date. Therefore, *B. bigelowii* could occur as a non-calcifying form^23^.

Our results revealed a marked contrast in the diversity patterns between diatoms and haptophytes (Fig. 3, Supplementary Fig. S2). At most of the sample sites, the diversity of diatoms was lower than that of haptophytes because diatom communities tended to be dominated by one or a few genera (i.e., lower evenness). The implication of this structure is that the most successful genera outcompeted the others due to intra-phylum competitive exclusion. It would also be caused by the ecophysiological traits of diatoms, which were developed to maximize their growth rates (i.e., *r*-strategists). For example, diatoms possess the efficient carbon fixation enzyme, Ribulose 1,5-bisphosphate carboxylase/oxygenase (RubisCO), and carbon concentration mechanisms (CCMs) for higher CO_2_ fixation capacity relative to other phytoplankton groups^49,50^. Another important trait for the success of diatoms is that large diatoms tend to have large vacuoles for the storage of nutrients^51^. As a consequence of these specializations, diatoms might have overlapping niches within a habitat with high water-column stability, which result in competitive exclusions within the group.

In contrast to diatoms, the haptophyte diversity remained fairly constant at most of the stations regardless of the sampling depth. The high levels of diversity were attributed to the coexistence of various taxa (i.e., high genus level evenness) rather than to high phylogenetic richness. The cluster analysis also revealed that the community compositions of haptophytes were relatively stable across the sampled sites, particularly in the tropical and subtropical regions (Supplementary Fig. S3). The diversity of haptophytes was negatively correlated with their biomass and nutrient concentrations (Fig. 4D, Table 1), suggesting that the haptophyte diversity might increase in low productive environments. Moreover, haptophytes are likely well-adapted to conditions of intermediate or low nutrient and turbulence (i.e., *K*-strategists)^18,25^. Many haptophyte species have adopted a mixotrophic lifestyle, and they are responsible for a large proportion of bacterivory in the ocean^52^. According to a theoretical study, mixotrophy is beneficial in oligotrophic environments, whereas specialization as autotrophs and heterotrophs is advantageous over mixotrophs in eutrophic environments^53,54^. Furthermore, some coccolithophores adapt to various nutritional conditions by possessing a heteromorphic life cycle, in which they alternate between a motile haploid and a non-motile diploid stage based on whether the environment is nutrient-limited or nutrient-replete, respectively^25^. Our results demonstrated that diverse non-calcifying haptophyte genera specialized as *K*-strategists were largely responsible for the abundance and diversity of haptophyte assemblages in the open ocean, although ecological success of the non-calcifying haptophyte groups has been poorly documented. Therefore, haptophytes might access multiple niches by diversifying the mode of nutrition and other aspects of physiology to reduce intra- and inter-phylum competition, and consequently, various haptophytes coexist in an environment. Overall, our study suggests that the difference in the ecological traits between diatoms and haptophytes, previously demonstrated at individual and metabolic levels, significantly affects the biogeography and diversity patterns of these phytoplankton.

The contrasting diversity pattern between diatoms and haptophytes changed in the subarctic and arctic regions (Fig. 3). As discussed earlier, these sites were characterized by relatively high nutrient availability (Table S1). Thus, preferential growth of various fast-growing diatom genera could contribute to the increased diversity. Similarly, higher diatom diversity was also observed around the equator, likely resulting from the nutrient enrichment by upwelling. On the other hand, the diversity of haptophytes decreased to some extent in the subarctic and polar ecosystems due to the preferential growth of specific genera, such as *Phaeocystis* and *Gephyrocapsa*/*Emiliania*. Some species in these genera are known to demonstrate *r*-selected properties and have the potential to form blooms in high latitudes^19,20^.

The diversity of diatoms was not monotonically correlated with any of the examined environmental variables or with the diatom abundance in either the surface or DCM layers (Table 1). However, with the exclusion of the stations in the neritic high latitude areas (i.e., 60–68°N along 170°W), significant unimodal (hump-shaped) relationships were detected between diatom diversity and temperature (Fig. 4A). These diversity patterns were consistent with that observed along a south-north transect in the Atlantic Ocean based on microscopic observations^8^, which also detected a unimodal relationship between phytoplankton diversity and sea surface temperature at a global scale based on satellite ocean color remote sensing data. In addition, Malviya *et al*.^10^ revealed that the diversity of diatoms tended to increase at low to middle latitudes (10°–30°) by using a large-scale dataset from the *Tara* Oceans expedition, although large variations existed among the sites. In our study, the haptophyte diversity also increased at mid-temperature region in both the surface and DCM layers, although the diversity remained high in the high temperature regions (Fig. 4C).

In conclusion, our results suggest that the distinct ecological strategies and physiological traits of diatoms and haptophytes underlie the shaping of the contrasting large-scale patterns in their abundance, diversity, and composition in the central Pacific Ocean. Our observations were conducted only during the summer period in the northern and southern hemispheres, but such basin-scale data are still very scarce. The phytoplankton abundance and diversity may fluctuate seasonally and will be affected by mesoscale eddies, cyclonic wind events, and a changing climate^2,15,55,56^. Therefore, continued efforts must be made to examine the spatio-temporal variations in the dynamics and diversity patterns of phytoplankton, which can alter the biogeochemical and ecosystem processes in the open ocean.

## Methods

### Sample collection

The field samplings were conducted at 35 stations in the western and central Pacific Ocean, the Bering Sea, and the Arctic Ocean during the KH-12-3 (from July 6 to August 14, 2012), KH-13-7 (from December 11, 2013 to February 12, 2014), and KH-14-3 (from June 23 to August 11, 2014) cruises of the R/V *Hakuho Maru* (JAMSTEC). Seawater samples were collected from the surface (0 to 10 m depth) and DCM layers using Niskin bottles attached to a CTD-RMS system, bucket, or laboratory research seawater collector. The depths of DCM layers were determined with an *in situ* chlorophyll fluorescence profile. The MLD was determined from CTD profiles following the method of Suga *et al*.^57^. The depth of euphotic layer (Z_eu_) was estimated from surface chlorophyll *a* concentration using the empirical formula given by Morel *et al*.^58^, or determined as the depth corresponding to 1% of the surface light intensity by *in situ* observation using Hyper Profiler (Satlantic)^59^. The hydrographic conditions at each sample site are shown in Supplementary Table 1.

### Nutrient analyses

Nutrient samples were collected into 10 mL plastic tubes and stored at −20 °C until analysis on land. The concentrations of nitrate plus nitrate, nitrite, phosphate, ammonia, and silicate were measured with a QuAAtro-2 continuous-flow analyzer (Gran + Luebbe).

### Real-time PCR

Water samples (1000 mL) for DNA analysis were collected sequentially on 10 μm pore size polycarbonate filters (Whatman, Maidstone, UK) without a vacuum and on 0.2 μm pore size polycarbonate Nuclepore membrane filters (Whatman) with gentle vacuum (<0.013 MPa). The samples were flash frozen in liquid nitrogen and stored in a deep freezer (−80 °C) until analysis. DNA extraction was performed following Endo *et al*.^60^, and the extracted DNA was purified using a NucleoSpin® gDNA Clean-up (Macherey-Nagel) following the manufacturer’s instructions.

The oligonucleotide primer sets specified for 18S rDNA fragments of diatoms and haptophytes were designed using the phytoplankton rDNA sequences retrieved from GenBank (http://www.ncbi.nlm.nih.gov) or were selected from previous studies^61,62^ with some modifications (Supplementary Table 3). The primer specificities for each phytoplankton clade were checked with the 18S rDNA database of SILVA TestPrime (http://www.arb-silva.de/search/testprime/). Standard curves for each 18S rDNA fragment were generated from duplicate serial dilutions of liner plasmids containing artificial gene fragments of the diatom, *Thalassiosira weissflogii* (accession number: AF374477), and the haptophyte, *Emiliania huxleyi* (accession number: M87327). Triplicate qPCR reactions were conducted for each assay using SYBR Premix Ex Taq II (Tli RNaseH Plus, Takara) with 0.4 µM primer concentrations and a Thermal Cycler Dice Real Time System (TP800, Takara). Both the reactions were initially incubated at 95 °C for 60 s, followed by 40 cycles at 95 °C for 5 s and then either at 55 °C (diatoms) or 54 °C (haptophytes) for 60 s.

The *nifH* fragments of the diazotrophic symbiont, UCYN-A1, were quantified based on a TaqMan assay with the primer and probe sets designed by Church *et al*.^22^ (Supplementary Table 4). We also used the primer and Taqman probe set designed against the UCYN-A1 host 18S rDNA fragment (Supplementary Table 4). Standard curves for each gene were generated from duplicate serial dilutions of linear plasmids containing artificial gene fragments obtained from the GenBank database with the following accession numbers: UCYN-A1 *nifH* (AF059642) and UCYN-A1 host 18S rDNA (KJ763100). The amplification reactions were performed using Premix Ex Taq (Perfect Real Time, TaKaRa) with 0.2 µM primers and 0.2 µM TaqMan probe. The thermal cycling condition for *nifH* gene quantification was as follows: 95 °C for 30 s, followed by 45 cycles at 95 °C for 5 s and at 60 °C for 30 s, whereas that for UCYN-A1 host 18S rDNA were as follows: 95 °C for 30 s, followed by 45 cycles at 95 °C for 5 s and at 57 °C for 30 s.

### Ion Torrent PGM sequencing

The V4 region of the 18S rDNA was amplified from the extracted DNA samples with barcoded fusion primer pairs targeted for diatoms and haptophytes, as described in Supplementary Table 3. The forward primers included the A-adaptor, key, and multiplex identifier (MID) sequences, whereas the reverse primers included the truncated Pi-adapter (trP1) sequence (see Table S3). Triplicate PCR amplifications were conducted for each sample using the TaKaRa Ex Taq Hot Start Version (TaKaRa). The PCR mixtures were prepared in a 25 μL total volume containing 1× Ex Taq Buffer, 0.2 mM dNTP, 0.4 μM of each primer, 0.625 unit of Taq polymerase, and 2 μL of template DNA. The thermal cycling conditions for diatoms were as follows: 94 °C hold for 60 s, followed by 30 cycles at 98 °C for 10 s, at 56 °C for 30 s, and at 72 °C for 60 s, and then a 72 °C hold for 10 min, whereas those for haptophytes were as follows: 94 °C hold for 60 s, followed by 30 cycles at 98 °C for 10 s, at 66 °C for 30 s, and at 72 °C for 60 s, and then a 72 °C hold for 10 min. We confirmed the success of PCR amplification by 1.5% agarose gel electrophoresis. The amplicons were purified using AMPure beads (Beckman Coulter) and then quantified with an Agilent 2100 Bioanalyzer using a DNA 1000 Kit (Agilent Technologies) following the manufacturer’s instructions. The PCR templates were then diluted to a final concentration of 13–26 pM and mixed in equal concentrations. Emulsion PCR was performed using an Ion One Touch^TM^ 2 system with Ion PGM^TM^ Template OT2 400 kit (Thermo Fisher Scientific) according to the manufacturer’s protocol. The products of emulsion PCR were enriched using an Ion One Touch^TM^ ES (Thermo Fisher Scientific) and then loaded onto an Ion 318^TM^ v2 chip. Sequencing of the amplicon libraries was performed using an Ion Torrent PGM system with the Ion PGM^TM^ sequencing 400 kit (Thermo Fisher Scientific) according to the manufacturer’s protocol.

### Sequence analyses

The sequences with polyclonal and no match against the A-adapter were initially filtered with Torrent Suite^TM^ Software (Thermo Fisher Scientific) and the data were exported as FASTQ files. The complete run files in each sample were deposited in the DDBJ Sequence Read Archive under accession numbers, DRA004899-DRA004901. Additional quality controls were performed using the FASTX-Toolkit software (http://hannonlab.cshl.edu/fastx_toolkit/). The sequencing reads were excluded based on the following criteria: (i) reads not containing the trP1 adapter sequence, and (ii) reads not containing the reverse primer sequence. The sequence regions between 18 and 270 bp for diatom libraries and between 18 and 440 bp for haptophyte libraries were used for downstream analysis. The reads that did not have a minimum 23 quality score over at least 80% of the sequence were also excluded. During these procedures, the forward and reverse primer sequences were trimmed off from all the reads.

Ten thousand reads for each library were used for the taxonomic assignment. Taxonomic classification was achieved using the SILVAngs web interface (https://www.arb-silva.de/ngs/) with >93% classification similarity to SILVA SSU Ref dataset 119. On average, 53.4% and 8.5% of the total sequences obtained were assigned to diatom taxa in the large and small fractions, respectively. On the other hand, averaged 95.9% of the total sequences obtained were assigned to haptophytes in the small fraction. The taxonomic assignments other than diatoms or haptophytes were removed from the classification results.

To check the robustness of the taxonomic classification, representative sequences obtained from the SILVAngs classification were also compared to the GenBank database using BLASTn (https://blast.ncbi.nlm.nih.gov). One of the representative sequences, which had the highest similarity score with each sequence in the GenBank database, was used for comparison of the classification results. If the results of the taxonomic classification were different from those obtained from SILVA analysis, we employed the results of the NCBI BLASTn. The haptophyte genera, *Gephyrocapsa* and *Emiliania*, were merged into a single taxon because they could not be distinguished from each other by their 18S rDNA sequence^63^. The putative novel haptophyte clades, such as OLI16010 and OLI6029, were also treated as independent taxa. The sequence reads that could not be classified down to genus level were assigned to order or subclass. The diversity of diatoms and haptophytes was assessed with the Shannon index (*H*′)^64^, based on genus-level taxonomic compositions.

### Statistical analyses

Statistical analyses were performed with the R statistical software (version 3.0.0) using the package vegan^65^. All the analyses were performed separately for the surface and DCM layers to avoid any effects derived from the sampling depth. To assess the relationships between algal abundance or diversity and environmental variables, linear regressions were analyzed using Pearson’s tests. Additionally, parabolic relationships were examined with a Mitchell-Olds and Shaw test (MOS test)^66^. The confidence levels for regressions were set at 95% (*p* < 0.05) and 99% (*p* < 0.01).

To determine the similarity within the phytoplankton communities, cluster analysis was used based on Bray-Curtis similarity and unweighted pair group method with arithmetic mean (UPGMA) linkage method. The groupings of phytoplankton community were verified by PERMANOVA^67^. The relationships between phytoplankton community compositions and environmental variables (i.e., temperature, salinity, nitrate, phosphate, and ammonia) were assessed with RDA. Since silicate had strong correlations with nitrate and phosphate (Pearson correlation, r = 0.78–0.91), we did not use this variable as a parameter of RDA to avoid multicollinearity in the multiple regression: redundant variables (those highly correlated with others) can lead to skewed or misleading results. Prior to ordination, detrended correspondence analysis (DCA)^68^ was used to calculate the length of the gradient (standard deviation, SD) of the environmental variables. We decided to use a liner response model (RDA) for ordination because the lengths of the gradient were less than 3 SD^69^. For RDA ordination, the community composition data were normalized with the Hellinger transformation^70^. To recognize the set of environmental parameters that could explain the variation in the phytoplankton community composition, the forward selection method was performed using the function ‘ordistep’ in the R package vegan.

### Data availability

Data generated during this study is included in this article.

## Electronic supplementary material

Supplementary Information
