# Supplementary material for: Contrasting biogeography and diversity patterns between diatoms and haptophytes in the central Pacific Ocean
Source: Sci Rep. 2018 Jul 19;8:10916. doi: 10.1038/s41598-018-29039-9 (PMC6053411; doi:10.1038/s41598-018-29039-9)
Supplement: Supplementary file 1 — Supplementary Information [file 41598_2018_29039_MOESM1_ESM.pdf]

Supplementary Information

Contrasting biogeography and diversity patterns between diatoms and haptophytes in the central Pacific Ocean

Hisashi Endo[1, 2, 3*], Hiroyuki Ogata[3], and Koji Suzuki[1, 2*]

[1]Faculty of Environmental Earth Science, Hokkaido University, North 10 West 5, Kita-ku, Sapporo, Hokkaido 060-0810, Japan

[2]CREST, Japan Science and Technology, North 10 West 5, Kita-ku, Sapporo, Hokkaido 060-0810, Japan

[3]Bioinformatics Center, Institute for Chemical Research, Kyoto University, Gokasho, Uji, Kyoto, 611-0011, Japan

**Corresponding authors*:

H. Endo (Email: endo@scl.kyoto-u.ac.jp, Phone: +81-774-38-3272)

K. Suzuki (Email: kojis@ees.hokudai.ac.jp, Phone/Fax: +81-11-706-2370)

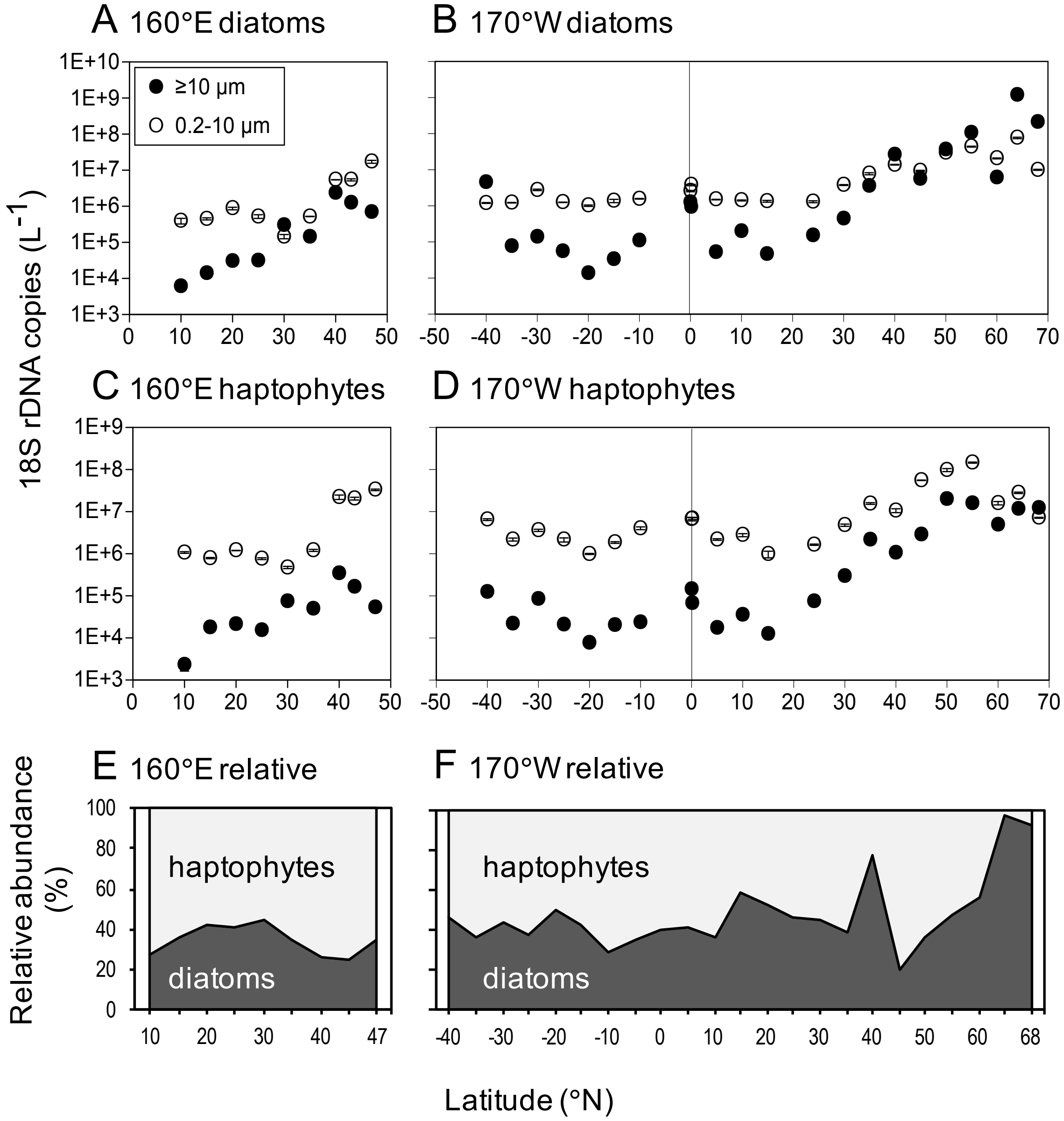

Figure S1. Meridional (South−North) variation in diatom and haptophyte abundances in the DCM layer. Upper (A and B) and middle (C and D) graphs show the abundance of size-fractionated 18S rDNA in diatoms and haptophytes, respectively. Left (A and C) and right (B and D) graphs indicate the abundance along the 160°E and 170°W transects, respectively. Error bars denote ± 1 standard deviation (SD, n = 3). Lower graphs (E and F) show the relative abundance of 18S rDNA from diatoms and haptophytes along the 160°E and 170°W transects, respectively.

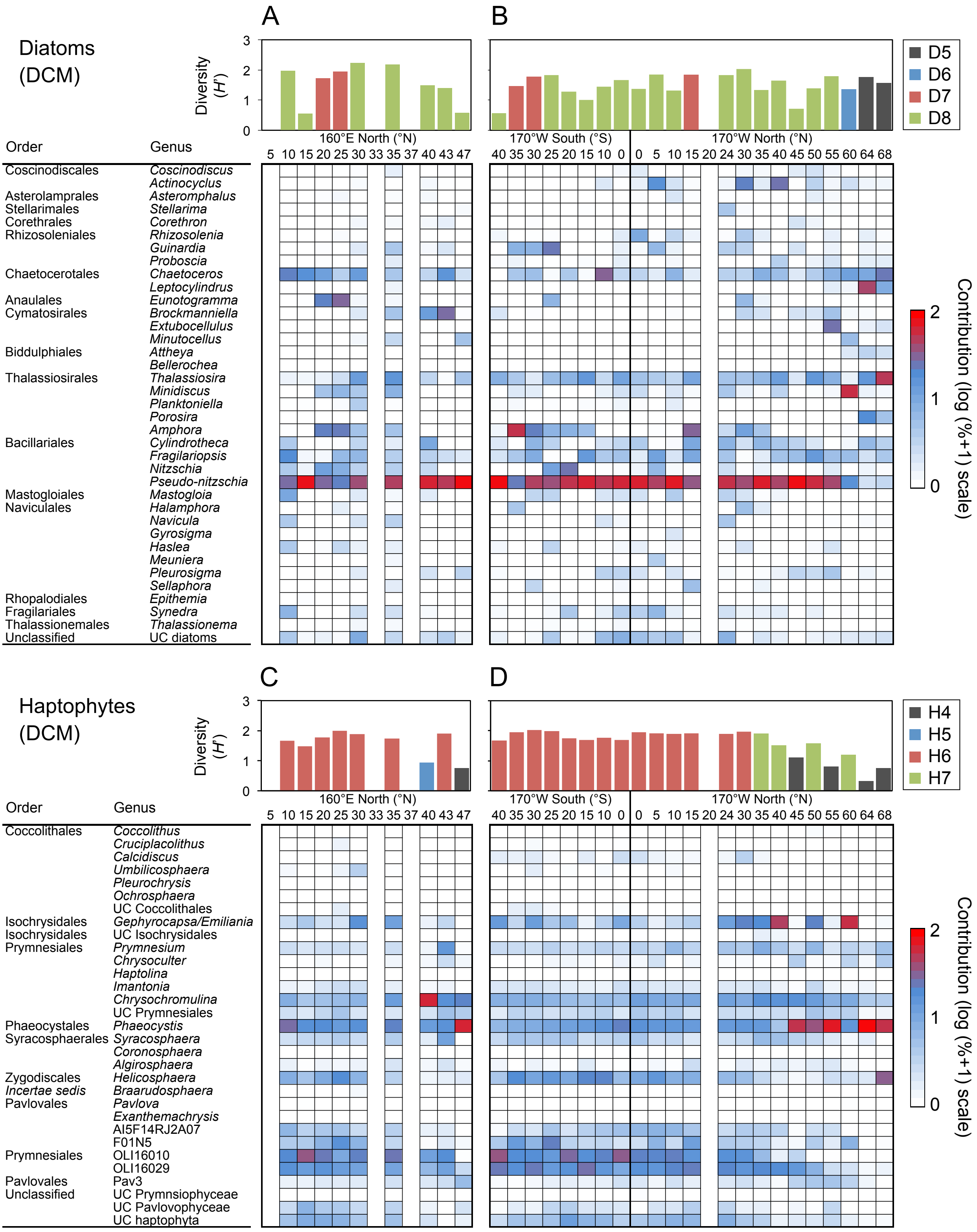

Figure S2. Diversity and distribution patterns of diatoms (A and B) and haptophytes (C and D) in the DCM layer. Left (A and C) and right (C and D) graphs represent the data collected along the 160°E and 170°W transects, respectively. Graphs for diatoms and haptophytes show data from the total (>0.2 μm) and small (0.2–10 μm) fractions, respectively. The scale bars are color-coded based on the biogeographical classification defined in Fig. S3. Diatom genera accounting for at least 1% of the total community in at least one of 66 sampling events are shown in the heatmaps.

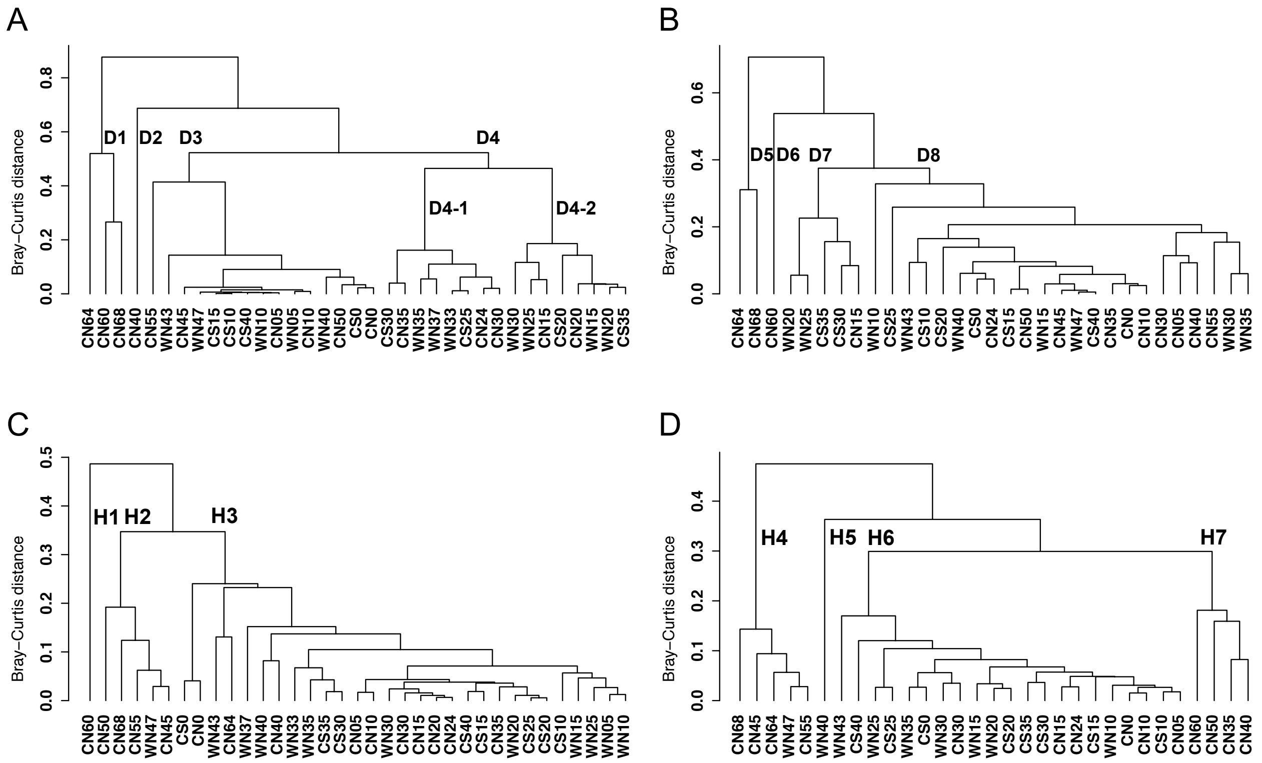

Figure S3. Cluster analysis based on diatom (A and B) and haptophyte (C and D) community compositions. Left (A and C) and right (B and D) graphs indicate the data from surface and DCM samples, respectively. The labels WN, CS, and CN indicate the samples collected from the western-north (KH-12-3), central-south (KH-13-7), and central-north (KH-14-3) Pacific. The following numbers represent sampling latitudes (°S or °N).

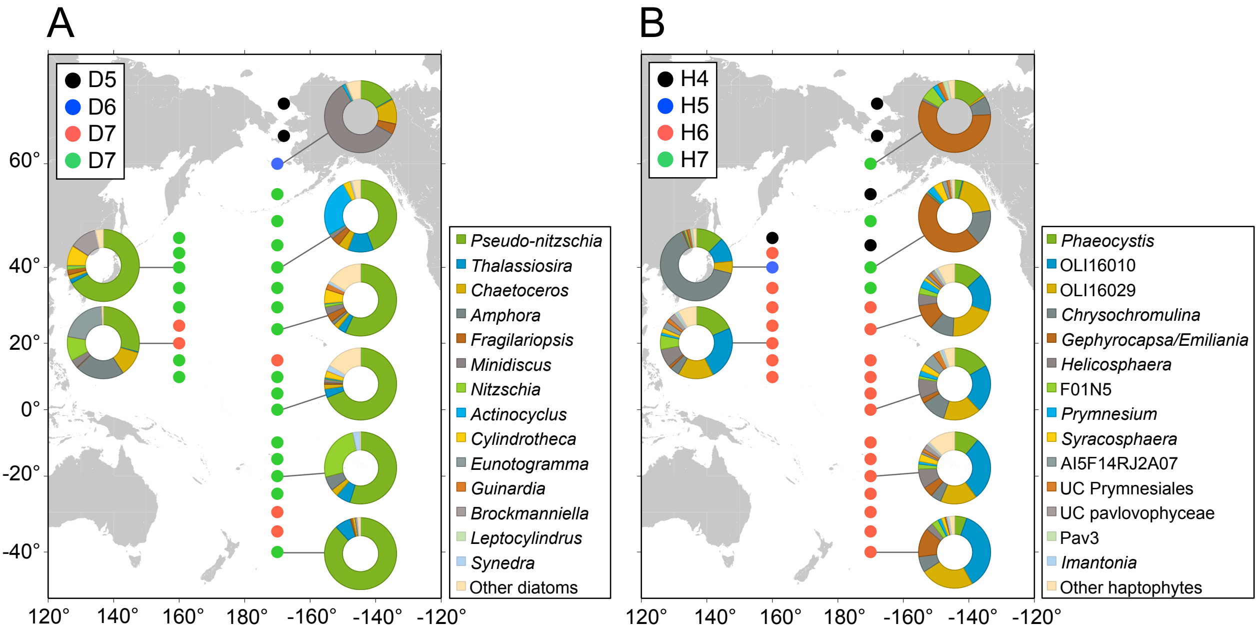

Figure S4. Biogeographical classifications of the study area based on the similarity of community compositions of diatoms (A) and haptophytes (B) in the DCM layer. Dots of identical color indicate locations of identical groups. Pie charts indicate the relative contribution of each genus to the total at 60°N and 20°N on the 160°W transect, and 60°N, 40°N, 24°N, 0°N, 20°S and 40°S on the 170°W transect. The base figure was created using Generic Mapping Tools (GMT) version 5.4.1 (https://www.soest.hawaii.edu/gmt/).

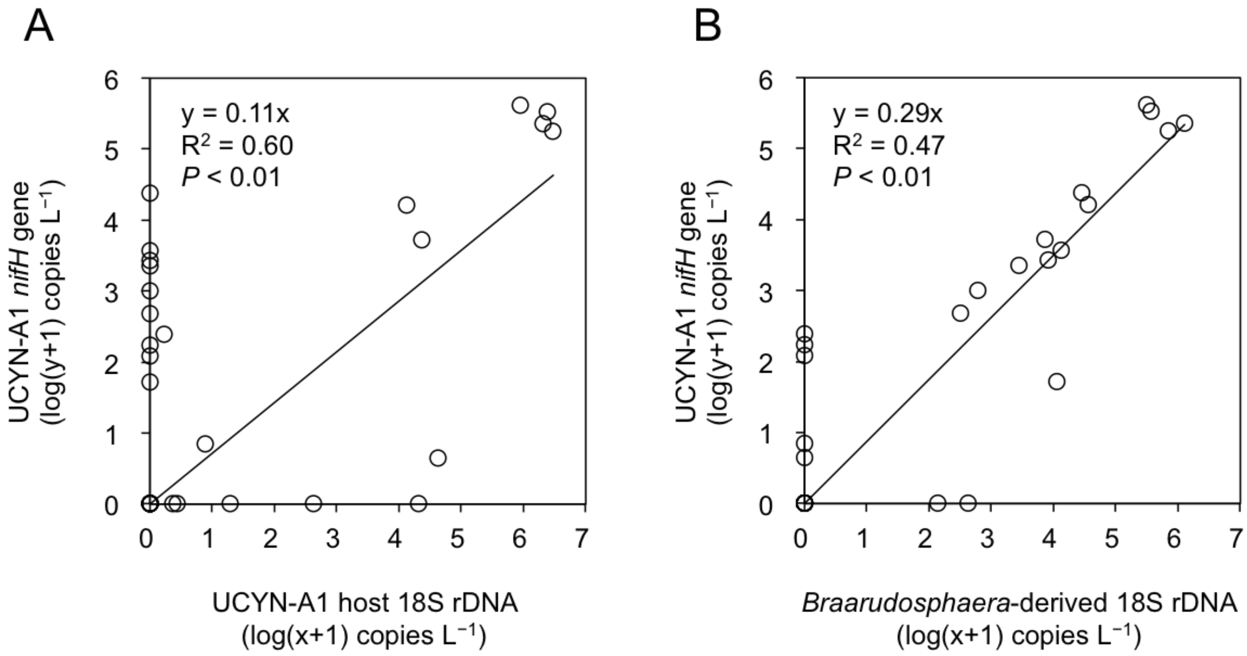

Figure S5. Correlations between (A) UCYN-A1 *nifH* and UCYN-A1 host 18S rDNA (Pearson's test, $p < 0.01$) and (B) UCYN-A1 *nifH* and *Braarudosphaera*-derived 18S rDNA (Pearson's test, $p < 0.01$) in the surface layer (n = 35). All variables were log-transformed for the figure. Note that the regression analysis was performed on non-log-transformed data.

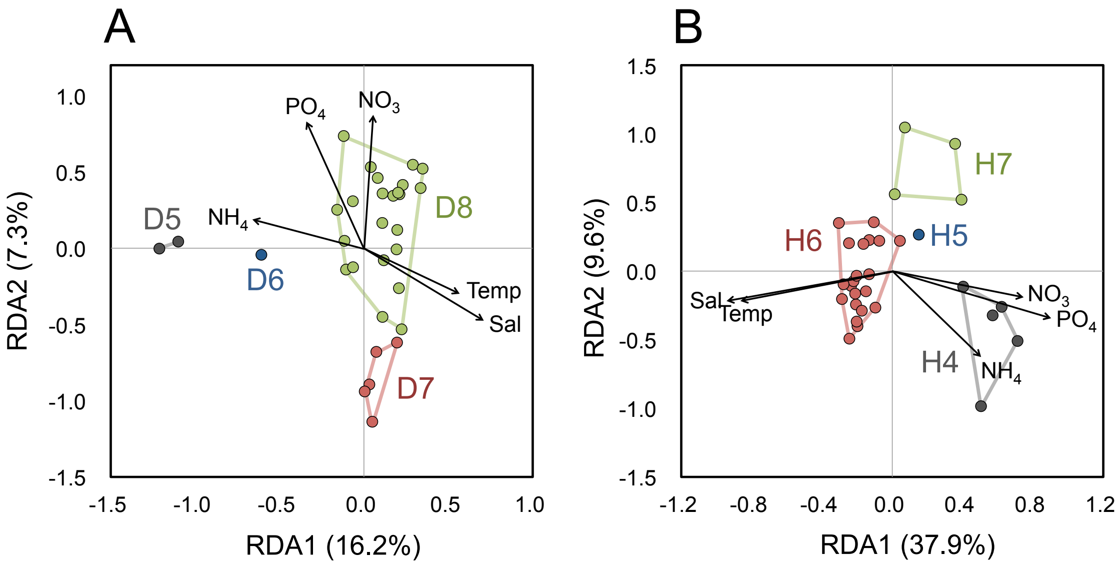

Figure S6. Redundancy analysis (RDA) ordinations for environmental variables and community compositions of diatoms (A) and haptophytes (B) in the DCM layer. The black arrows indicate the vectors of the explanatory variables. The sample events contained in each cluster (Supplementary Fig. S3) are distinguished by different colors.

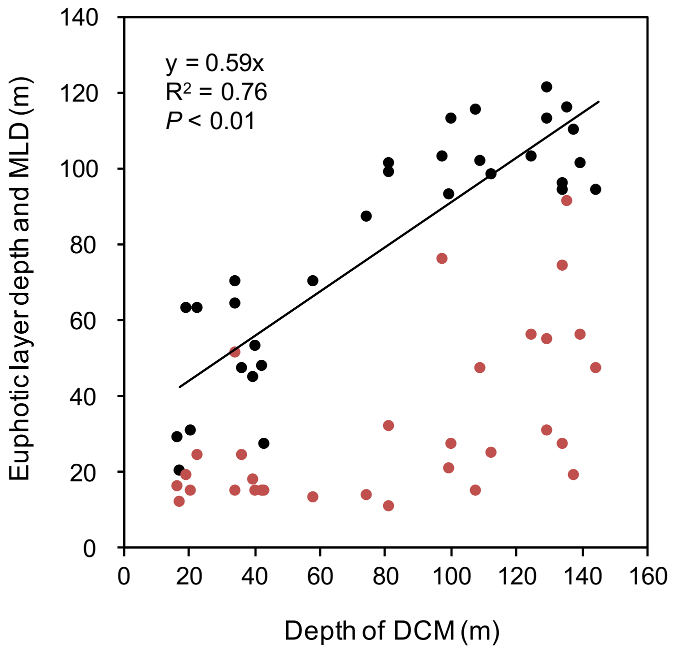

Figure S7. Correlations between the DCM depth and euphotic layer depth (black dot) or MLD (red dot) across stations (n = 31). Singnificant correlation was detected only between the depths of DCM and euphotic layers ($r^2 = 0.76$, $p < 0.01$).

Table S1. Environmental and biological parametrs in the (A) surface and (B) DCM layers across stations.

(A)

Cruise	St	Date	Lat (N)	Long (E)	SFD (m)	MLD (m)	Z_{eu} (m)	Sampling depth (m)	Temp (°C)	Sal	Chl *a* (µg L^{-1})	Nutrients (µmol L^{-1})			
												NO_3	NH_4	PO_4	$Si(OH)_4$
KH-12-3	1	10 Jul.	46°56’	159°59’	5156	15	48	5	8.7	32.89	0.41	6.05	0.81	0.74	11.80
	2	11 Jul.	43°30’	160°00’	5327	24	47	5	14.1	33.87	0.39	7.81	0.68	0.92	10.63
	3	13 Jul.	40°00’	160°00’	5506	18	45	5	17.3	34.32	0.44	0.38	0.07	0.16	4.97
	4	14 Jul.	37°00’	160°00’	4081	23	81	0	21.7	34.50	0.12	0.00	0.13	0.05	2.02
	5	15 Jul.	35°00’	160°00’	4531	14	87	5	24.0	34.51	0.10	0.00	0.21	0.03	2.20
	6	16 Jul.	33°00’	160°00’	4585	14	85	0	24.4	34.70	0.09	0.00	0.07	0.03	2.44
	7	17 Jul.	30°01’	159°59’	5687	11	99	5	27.3	34.84	0.06	0.00	0.29	0.02	2.12
	8	18 Jul.	25°00’	160°00’	5748	19	110	5	29.1	35.40	0.03	0.00	0.96	0.02	1.59
	9	20 Jul.	20°00’	159°59’	4423	56	103	5	29.2	34.96	0.07	0.00	0.02	0.02	1.52
	10	22 Jul.	15°00’	160°00’	5376	56	101	5	29.2	34.91	0.05	0.00	0.00	0.07	1.62
	11	23 Jul.	10°00’	160°00’	4030	55	113	5	29.2	34.40	0.04	0.00	0.07	0.09	1.70
	12	24 Jul.	5°00’	160°00’	4009	30	85	5	28.9	34.74	0.09	0.00	0.00	0.12	1.72
KH-13-7	1	23 Dec.	0°00’	-170°00’	5459	15	53	5	27.3	35.49	0.34	5.59	0.09	0.56	2.56
	3	26 Dec.	-10°00’	-170°00’	4968	47	102	5	30.4	35.13	0.06	3.77	0.17	0.53	1.81
	4	2 Jan.	-15°00’	-170°04’	4772	27	96	10	29.1	35.26	0.07	0.00	0.00	0.21	1.23
	5	4 Jan.	-20°01’	-170°02’	5333	47	94	5	27.8	35.43	0.08	0.00	0.00	0.14	1.15
	6	7 Jan.	-25°00’	-170°01’	5679	25	98	5	26.3	35.55	0.08	0.00	0.02	0.06	0.98
	7	9 Jan.	-30°00’	-170°00’	5389	15	115	5	24.0	35.44	0.06	0.00	0.08	0.05	0.99
	8	11 Jan.	-35°00’	-170°00’	5207	31	121	6	21.7	35.28	0.04	0.00	0.00	0.08	1.04
	9	13 Jan.	-40°00’	-170°00’	4627	32	101	5	19.8	35.07	0.08	0.00	0.00	0.15	1.13
KH-14-3	1	4 Jul.	0°17’	-170°03’	5418	51	70	5	28.3	35.40	0.28	5.09	0.00	0.60	1.43
	2	5 Jul.	4°58’	-169°55’	5700	74	94	5	29.3	34.23	0.07	0.04	0.00	0.21	0.31
	3	6 Jul.	10°02’	-170°07’	4458	76	103	5	28.5	34.77	0.08	0.02	0.00	0.25	0.35
	4	8 Jul.	15°01’	-170°02’	5653	91	116	5	27.5	34.99	0.06	0.02	0.00	0.20	0.37
	5	9 Jul.	20°01’	-169°55’	2493	40	100	5	27.5	35.16	0.05	0.06	0.00	0.18	0.05
	6	10 Jul.	24°05’	-170°01’	4669	27	113	5	26.5	35.57	0.05	0.01	0.01	0.08	0.24
	7	20 Jul.	30°02’	-170°00’	5460	21	93	5	26.0	35.23	0.06	0.02	0.00	0.05	1.47
	8	21 Jul.	35°02’	-170°01’	5812	13	70	5	23.8	34.59	0.07	0.02	0.00	0.07	2.50
	9	23 Jul.	40°02’	-170°00’	5421	24	63	5	16.7	33.71	0.22	0.46	0.00	0.22	2.39
	10	24 Jul.	45°03’	-170°04’	5941	15	64	5	12.8	33.00	0.21	12.60	0.20	1.33	20.74
	11	25 Jul.	50°00’	-170°02’	5344	19	63	5	11.6	32.56	1.11	10.30	0.01	1.29	26.34
	12	27 Jul.	55°00’	-170°00’	2909	15	31	5	11.4	32.60	1.10	1.43	0.06	0.84	3.52
	13	28 Jul.	60°00’	-170°40’	67	16	29	5	9.8	30.44	0.46	0.07	0.05	0.44	2.68
	14	29 Jul.	64°15’	-168°00’	37	12	20	5	9.1	31.16	2.21	0.15	0.09	0.35	0.00
	15	30 Jul.	68°00’	-168°00’	54	15	27	5	4.1	32.32	0.21	1.09	1.46	0.81	0.14

(B)

Cruise	Station	Date	Lat (N)	Long (E)	SFD (m)	MLD (m)	Z_{eu} (m)	Sampling depth (m)	Temp (°C)	Sal	Chl *a* (µg L^{-1})	Nutrients (µmol L^{-1}) NO_3	NH_4	PO_4	$Si(OH)_4$
KH-12-3	1	10 Jul.	46°56’	159°59’	5156	15	48	43	2.9	33.17	1.06	17.97	1.52	1.59	20.26
	2	11 Jul.	43°30’	160°00’	5327	24	47	37	10.9	33.78	0.53	7.83	0.70	0.52	10.65
	3	13 Jul.	40°00’	160°00’	5506	18	45	40	12.7	34.26	0.48	6.43	0.63	0.59	13.16
	5	15 Jul.	35°00’	160°00’	4531	14	87	75	19.0	34.67	0.34	0.76	0.12	0.11	3.17
	7	17 Jul.	30°01’	159°59’	5687	11	99	82	18.2	34.77	0.47	1.14	0.09	0.13	3.13
	8	18 Jul.	25°00’	160°00’	5748	19	110	138	18.3	34.79	0.26	1.89	0.00	0.16	3.44
	9	20 Jul.	20°00’	159°59’	4423	56	103	125	23.1	35.17	0.31	0.13	0.00	0.05	2.07
	10	22 Jul.	15°00’	160°00’	5376	56	101	140	25.8	35.19	0.34	0.00	0.00	0.04	1.60
	11	23 Jul.	10°00’	160°00’	4030	55	113	130	25.2	34.99	0.30	0.32	0.00	0.16	2.05
KH-13-7	1	23 Dec.	0°00’	-170°00’	5459	15	53	41	27.2	35.49	0.33	5.80	0.17	0.58	2.59
	3	26 Dec.	-10°00’	-170°00’	4968	47	102	110	26.4	35.99	0.28	0.00	0.00	0.20	1.06
	4	2 Jan.	-15°00’	-170°04’	4772	27	96	135	25.2	36.15	0.30	0.90	0.03	0.35	1.29
	5	4 Jan.	-20°01’	-170°02’	5333	47	94	145	23.3	35.60	0.27	0.00	0.00	0.13	1.02
	6	7 Jan.	-25°00’	-170°01’	5679	25	98	113	21.0	35.59	0.39	1.25	0.04	0.20	1.10
	7	9 Jan.	-30°00’	-170°00’	5389	15	115	108	17.2	35.48	0.49	0.57	0.07	0.19	1.33
	8	11 Jan.	-35°00’	-170°00’	5207	31	121	130	15.7	35.30	0.34	1.02	0.00	0.24	1.38
	9	13 Jan.	-40°00’	-170°00’	4627	32	101	82	13.9	35.11	0.37	1.93	0.00	0.32	1.46
KH-14-3	1	4 Jul.	0°17’	-170°03’	5418	51	70	35	28.2	35.41	0.32	5.42	0.00	0.67	1.52
	2	5 Jul.	4°58’	-169°55’	5700	74	94	135	25.9	34.88	0.23	2.51	0.00	0.44	2.26
	3	6 Jul.	10°02’	-170°07’	4458	76	103	98	24.5	34.72	0.28	0.14	0.09	0.30	0.76
	4	8 Jul.	15°01’	-170°02’	5653	91	116	136	22.7	35.18	0.22	0.44	0.00	0.24	1.05
	6	10 Jul.	24°05’	-170°01’	4669	27	113	101	19.5	35.13	0.26	0.02	0.00	0.09	1.56
	7	20 Jul.	30°02’	-170°00’	5460	21	93	100	16.8	34.75	0.44	0.59	0.03	0.16	3.60
	8	21 Jul.	35°02’	-170°01’	5812	13	70	59	16.1	34.61	0.50	0.68	0.00	0.16	3.81
	9	23 Jul.	40°02’	-170°00’	5421	24	63	23	13.7	34.18	0.28	2.80	0.13	0.36	6.48
	10	24 Jul.	45°03’	-170°04’	5941	15	64	35	11.4	33.03	0.27	13.96	0.05	1.53	23.13
	11	25 Jul.	50°00’	-170°02’	5344	19	63	20	11.4	32.56	0.85	11.00	0.01	1.50	25.57
	12	27 Jul.	55°00’	-170°00’	2909	15	31	21	8.9	32.72	1.11	15.92	0.58	1.74	29.54
	13	28 Jul.	60°00’	-170°40’	67	16	29	17	4.4	30.53	0.60	0.49	0.36	0.71	2.27
	14	29 Jul.	64°15’	-168°00’	37	12	20	18	5.5	31.38	10.03	1.42	0.62	0.83	4.03
	15	30 Jul.	68°00’	-168°00’	54	15	27	44	2.6	32.57	2.45	8.25	6.18	1.74	7.94

Abbreviations: St, station; Long, longitude; Lat, latitude; SFD, seafloor depth; MDL, mixed layer depth; Z_{eu}, euphotic layer depth; Temp, temperature; Sal, salinity; Chl *a*, chlorophyll *a*; NO_3, nitrate; NH_4, ammonia; PO_4, phosphate; $Si(OH)_4$, Silicate.

Table S2. Representative clades and average contributions in each cluster. The abbreviation UC indicates unclassified (i.e., sequence reads could not be classified to genus).

Class	Depth	Cluster	No. of samples	Representative clades (average contribution to the total) 1st	2nd	3rd	4th
Diatoms	Surface	D1	3	*Chaetoceros* (35.4%)	*Leptocylindrus* (29.8%)	*Minidiscus* (12.6%)	*Thalassiosira* (8.6%)
		D2	1	*Actinocyclus* (72.0%)	*Pseudo-nitzschia* (13.5%)	*Thalassiosira* (6.2%)	*Fragilariopsis* (5.4%)
		D3	15	*Pseudo-nitzschia* (75.4%)	*Fragilariopsis* (4.0%)	*Extubocellulus* (3.5%)	*Chaetoceros* (3.4%)
		D4-1	8	*Nitzschia* (53.3%)	*Pseudo-nitzschia* (23.9%)	*Mastogloia* (9.0%)	*Cylindrotheca* (5.5%)
		D4-2	8	*Mastogloia* (55.7%)	*Pseudo-nitzschia* (27.9%)	*Nitzschia* (5.9%)	*Rhizosolenia* (3.5%)
	DCM	D5	2	*Thalassiosira* (32.8%)	*Leptocylindrus* (25.9%)	*Chaetoceros* (16.5%)	*Porosira* (11.3%)
		D6	1	*Minidiscus* (58.6%)	*Pseudo-nitzschia* (16.7%)	*Chaetoceros* (11.1%)	*Minutocellus* (4.7%)
		D7	5	*Pseudo-nitzschia* (31.4%)	*Amphora* (30.8%)	*Eunotogramma* (10.2%)	*Fragilariopsis* (5.1%)
		D8	23	*Pseudo-nitzschia* (59.1%)	*Chaetoceros* (6.3%)	*Thalassiosira* (5.8%)	*Fragilariopsis* (4.2%)
Haptophytes	Surface	H1	1	*Gephyrocapsa/Emiliania* (47.9%)	UC Prymnesiales (18.5%)	*Chrysochromulina* (12.2%)	F01N5 (9.1%)
		H2	5	*Phaeocystis* (54.9%)	*Chrysochromulina* (11.5%)	OLI16029 (6.1%)	*Gephyrocapsa/Emiliania* (5.6%)
		H3	29	OLI16029 (18.0%)	*Chrysochromulina* (17.3%)	*Phaeocystis* (11.8%)	*Prymnesium* (10.3%)
	DCM	H4	5	*Phaeocystis* (69.3%)	*Chrysochromulina* (10.0%)	*Helicosphaera* (7.2%)	OLI16029 (3.6%)
		H5	1	*Chrysochromulina* (64.8%)	*Phaeocystis* (12.4%)	OLI16010 (11.0%)	OLI16029 (5.5%)
		H6	21	OLI16010 (21.6%)	OLI16029 (16.3%)	*Phaeocystis* (14.7%)	*Helicosphaera* (9.3%)
		H7	4	*Gephyrocapsa/Emiliania* (36.4%)	*Phaeocystis* (17.4%)	*Chrysochromulina* (13.3%)	OLI16029 (11.4%)

Table S3. Primer pairs used for SYBR green-based qPCR and NGS assays targeting 18S rRNA gene of diatoms and haptophytes.

Experiments/Primer name	Sequence (5' to 3')	References
**qPCR**		
Diatom-specific 18S rRNA gene forward	AACTACTGCGAAAGCATTTA	This study
Diatom-specific 18S rRNA gene reverse	GACTACGATGGTATCTRATCAT	Modified from Zimmermann et al. (2011)
Haptophyte-specific 18S rRNA gene forward	TCAGGGGCACTCGTATTC	This study
Haptophyte-specific 18S rRNA gene reverse	GATCAGTGAAAACATCCCTGG	Egge et al. (2013)
**NGS**		
18S rRNA gene universal forward	AGCCGCGGTAATTCCA*	This study
Diatom-specific 18S rRNA gene reverse	GACTACGATGGTATCTRATCAT**	Modified from Zimmermann et al. (2011)
Haptophyte-specific 18S rRNA gene reverse	GATCAGTGAAAACATCCCTGG**	Egge et al. (2013)

* This primer included the A-adaptor (5’-CCATCTCATCCCTGCGTGTCTCCGAC-3’), key (5’-TCAG-3’) and multiplex identifier (MID) sequences set by the manufacturer (Thermo Fisher Scientific) at the 5’ terminus.

** This primer included the truncated Pi-adapter (trP1: 5’-CCTCTCTATGGGCAGTCGGTGAT-3’) sequence at the 5’ terminus.

Table S4. Primer and probe sequences used for Taqman-based qPCR assays targeting *nifH* and 18S rRNA gene.

Experiments/Primer name	Sequence (5' to 3')	References
UCYN-A1 *nifH* forward	AGCTATAACAACGTTTTATGCGTTGA	Church et al. (2005)
UCYN-A1 *nifH* reverse	ACCACGACCAGCACATCCA	Church et al. (2005)
UCYN-A1 *nifH* probe	TCTGGTGGTCCTGAGCCTGGA	Church et al. (2005)
UCYN-A1 host 18S rRNA gene forward	AGGTTTGCCGGTCTGCCGAT	J. P. Zehr (personal communication)
UCYN-A1 host 18S rRNA gene reverse	ATCCGTCTCCGACACCCGCTC	J. P. Zehr (personal communication)
UCYN-A1 hpst 18S rRNA gene probe	CTGGTAGAACTGTCCTTCC	J. P. Zehr (personal communication)

SI References

Zimmermann, J., Jahn, R. & Gemeinholzer, B. Barcoding diatoms: evaluation of the V4 subregion on the 18S rRNA gene, including new primers and protocols. *Org. Divers. Evol.* **11**, 173–192 (2011).

Egge, E. S., Eikrem, W., & Edvardsen, B. Deep-branching Novel Lineages and High Diversity of Haptophytes in the Skagerrak (Norway) Uncovered by 454 Pyrosequencing. *J. Euk. Microbiol.* **62**, 121–140 (2015).

Church, M. J., Jenkins, B. D., Karl, D. M. & Zehr, J. P. Vertical distributions of nitrogen-fixing phylotypes at Stn ALOHA in the oligotrophic North Pacific Ocean. *Aquat. Microbiol. Ecol.* **38**, 3–14 (2005).
